# Protocol to build open-source Discrimin8 maze to study discrimination of reward-context associations in mice during open-field foraging

**DOI:** 10.1016/j.xpro.2026.104415

**Published:** 2026-03-05

**Authors:** Gergely Tarcsay, Laura A. Ewell

**Affiliations:** 1 Anatomy & Neurobiology, School of Medicine, University of California, Irvine, Irvine, CA, USA; 2 Neurobiology of Brain and Behavior, Charlie Dunlop School of Biological Sciences, University of California, Irvine, Irvine, CA, USA; 3 Center for Learning and Memory, University of California, Irvine, Irvine, CA, USA

**Keywords:** neuroscience, behavior, computer sciences

## Abstract

The hippocampus is known to process context-specific memories. We provide a protocol for studying context discrimination in mice utilizing the open-source Discrimin8 maze. We describe the steps for building the maze and using custom-written codes implementing the task. Finally, instructions are provided to train mice on the task. This protocol implements a low-cost, automated maze allowing for investigation of context discrimination in freely moving mice.

For complete details on the use and execution of this protocol, please refer to Tarcsay et al.^1^

## Highlights

• Instructions to build the Discrimin8 maze
• Guidance to calibrate the custom-written codes to control the maze
• Steps for testing context discrimination in a discrimination/generalization task

## Abstract (editor highlights)

Publisher’s note: Undertaking any experimental protocol requires adherence to local institutional guidelines for laboratory safety and ethics.

## Graphical abstract

**Figure:**
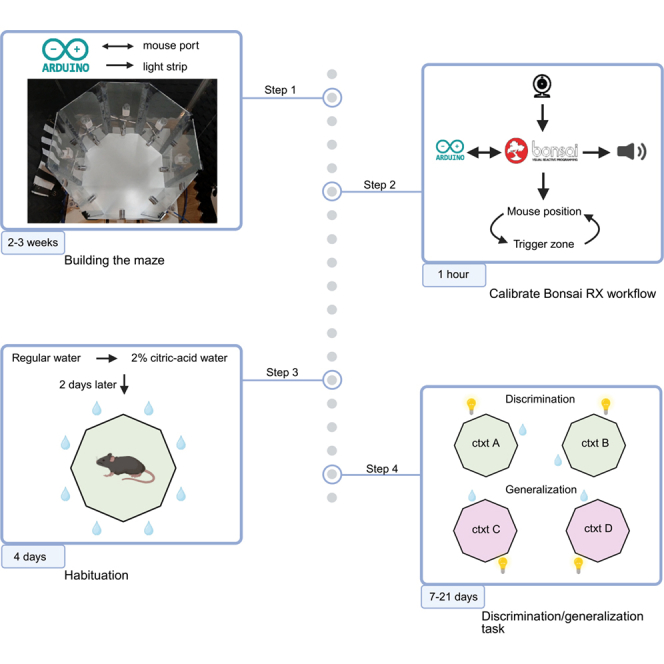

## Before you begin

The hippocampus is known to encode different contexts, potentially contributing to creating context-specific memories. Several experiments have been designed to investigate hippocampal context codes while rodents were exposed to distinct contexts,^2^^,^^3^^,^^4^^,^^5^ however, it is unknown how different cognitive demands, such as the relevance of context, impact hippocampal computations.

We developed the open-source Discrimin8 maze and designed a discrimination/generalization task in which the relevance of context may be manipulated by the experimenter. Our task allows the experimenter to directly compare context-discrimination under two distinct behavioral conditions: 1) in the discrimination paradigm, mice must discriminate the contexts to retrieve reward at distinct locations, therefore the context has high behavioral relevance 2) in the generalization paradigm reward retrieval is not tied to context, therefore the context has low behavioral relevance. This protocol describes how to build the Discrimin8 maze and provides instructions to train mice on the discrimination/generalization task.

### Innovation

Existing context discrimination tasks are typically not suitable to assess behavioral relevance^2^^,^^3^ or are implemented in a head-fixed paradigm.^4^^,^^5^ Moreover, other automated tasks may have high purchasing burden. This protocol describes a low-cost, open-source automated maze to investigate context discrimination in freely moving mice, inspired by Morales et al.^6^ Importantly mice forage for a hidden trigger zone in each context they are exposed to, therefore, if combined with recording, the experimenter can construct spatial maps for each context and test hypothesis about context-dependent spatial coding.

### Institutional permissions

All animal experiments were performed as approved by the Institutional Animal Care and Use Committee at the University of California, Irvine. To perform animal experiments described in this protocol, prior approval according to institutional or state legislation is required.

### Preparation of the maze

**Timing: 2–3 weeks**

Proper setup of the Discrimin8 maze is essential for successful experiments. The walls of the maze are laser-cut, transparent plexiglass. A key component of the maze is the reward port (Sanworks), containing an infrared (IR) emitter and sensor allowing to track nose pokes, an LED that may be used as a visual guide during training, and a solenoid valve for liquid delivery. Each wall is equipped with a reward port and a light strip.

1. Assemble the arena:
  a. Cut a 55 cm X 55 cm piece from a non-slip, white material that will serve as the floor. We have found that the top of an IKEA LACK side table works well and does not require cutting.
  b. Attach the 8 plexiglass walls (38 × 18 cm) to each other and to the floor using a hot glue gun. Application of additional support such as corner braces with 135 degree angles (octagon interior angles) is recommended. Each wall has a circular hole (diameter is 2.3 cm) 1.5 cm high from the floor (see key resources table for more details and link to design files).
  c. Guide the reward port through the hole on the wall and use double-sided tape to hold it in position.
  d. Assemble the liquid container:
    i. Take a 10 mL syringe and remove the plunger.
    ii. Connect the syringe to a three-way stop cock.
    iii. Connect the stop cock to the open end of the solenoid valve.
    iv. Reinforce the syringe against the wall (e.g., with transparent tape).
  e. Prepare the light strips:
    i. Cut 10 cm long light strips at the positive/negative terminals.
    ii. Cut a rainbow cable to the desired length.
    iii. Strip the isolation 1–2 cm long on one end of the cable.
    iv. Apply a small amount of soldering paste on the surface of the positive terminal of the light strip.
    v. Using a soldering iron, apply a drop of solder on the positive terminal and the exposed end of the cable until a smooth metal surface is formed between them.
    vi. Strip the isolation 2–3 cm long on the other end of the cable and connect a male header pin to the cable and add plastic housing.
    vii. Repeat steps ii-vi. for the negative terminal.
    viii. Repeat steps i-vii. for each light strip (x8).
  f. Tape the light cues to the outer side of the walls in a diagonal orientation such that the light faces the arena, approximately 10 cm above the reward port.
  g. Build a metal frame in the shape of a cube for the maze housing utilizing T-Slotted aluminum extrusion (dimensions should be roughly 70 cm x 70 cm x 70 cm).
  h. Slot in foam board to the sides of the frame.
  i. Cover the walls and doors with soundproof foam inside the cube. This helps isolate tones in the maze if you build the maze near other experiments.
  j. Mount a camera on the ceiling of the box, centered above the maze.
  k. Place two speakers near the maze (e.g., left and right side of the maze) and within the cube (to minimize sound spread to the rest of the lab).
  l. Place distal cues around the arena on the interior walls of the cube.

**CRITICAL:** When using dark fur mice, the floor must be white or a very light color as the real-time tracking relies on the contrast between the floor and the mouse. For using white mice on white floor, please see the troubleshooting section.

**Figure 1:**
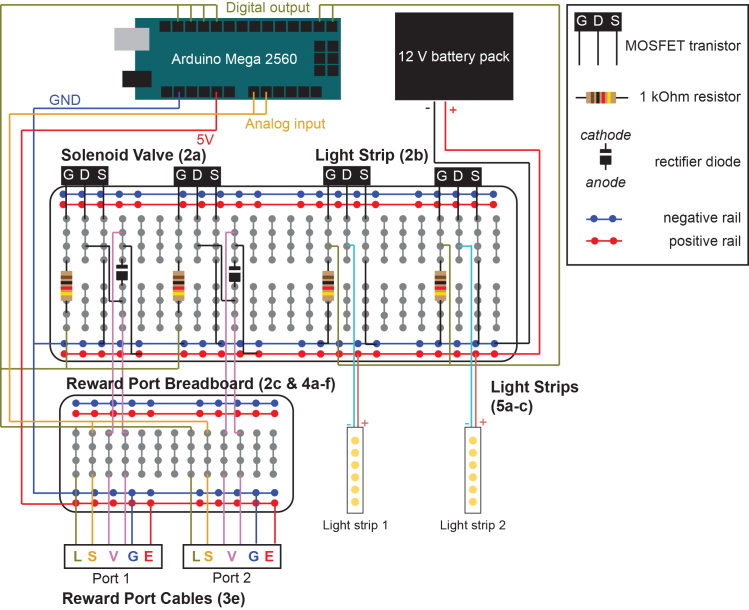
Electrical circuit diagram for two reward ports and two light strips Solenoid valve and light strip circuits are built on a breadboard that is connected to an Arduino Mega 2560 and a 12 V battery pack. Components of the reward port, such as LED (L), IR sensor (S), solenoid valve (V), ground (G) and IR emitter (E) are connected to the Arduino. Notation such as ‘2a, 2b, etc’ refers to steps in the ‘preparation of the maze’ section.

**Figure 2:**
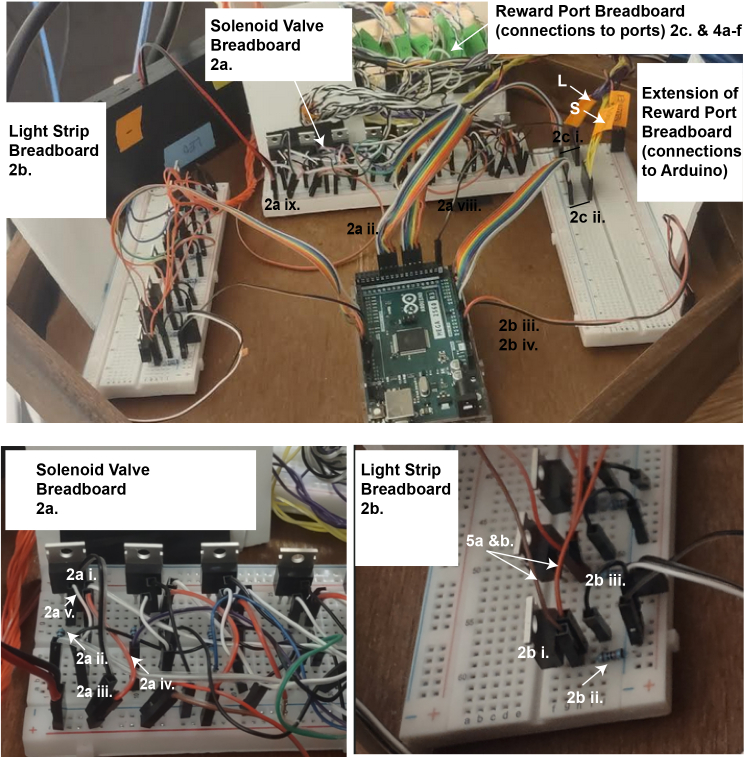
Electrical circuit image for full 8 port, 8 light strip maze Real world image of the circuit that corresponds to the diagram in Figure 1. Note that the Solenoid Valve and Light Strip breadboards are now on two rather than one breadboard as depicted in Figure 1. Furthermore the Reward Port Breadboard has been expanded to two: one in the back that handles the incoming cables (see Figure 3) and one on the right that connects reward port signals to the Arduino. Below, blown up pictures of the Solenoid Valve and Light Strip circuit are shown with individual components indicated. Again, the notation throughout (Number, letter) refers to the ‘preparation of the maze’ section.

**Figure 3:**
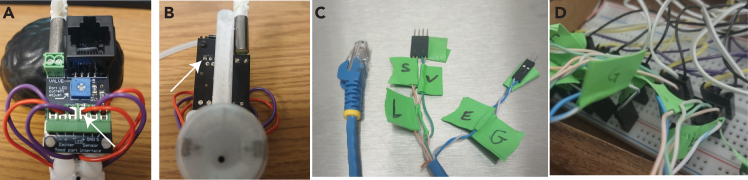
Screw terminals of the different components of the reward port (A) *Bpod interface* in the back of a reward port. As an example, the white arrow indicates the location of the screw terminal of *LED (+)*. Note that screw terminals of the solenoid valve (*VAVLE*) are located on the top of the interface. (B) The front side of the *Bpod interface*. White arrow marks the terminal that corresponds to *Emitter (+)*. (C) A split and mapped ethernet cable is shown. It was plugged into the jack in A to perform the mapping. (D) An image of reward port connections from the ethernet cable plugged into the ‘reward port breadboard’ seen in Figure 2.

2. Build the electrical circuit.
  a. Wire the breadboard for the solenoid valve:
    i. Connect a MOSFET transistor to the breadboard.
    ii. Connect the gate terminal (G) of the MOSFET to a digital output pin of the Arduino via a 1 kOhm resistor (Figure 1, olive green).
    iii. Connect the source terminal (S) of the MOSFET to the negative rail of the breadboard.
    iv. Connect a rectifier diode to the breadboard. The diode cathode should be connected to the positive rail of the breadboard.
    v. Connect the drain terminal (D) of the MOSFET to the diode anode.
    vi. Connect the diode anode and cathode to a separate breadboard that will eventually be wired to the reward ports which will refer to as the reward port breadboard (Figure 1, magenta). Other components of the reward port breadboard are described in 2c and the connections to the reward ports are described in steps 3 and 4.
    vii. Repeat steps i-vi. for each solenoid valves (x8).
    viii. Connect the negative rail of the breadboard to the Arduino ground (GND) pin.
    ix. Connect the 12 V battery box to the positive and negative rails of the breadboard.
      ***Note:*** A circuit diagram (Figure 1) and a picture of the full circuit (Figure 2) are provided. The diagram in Figure 1 shows two ports for simplicity and also shows only two breadboards. When building up the full circuit – we it is useful to utilize four breadboards (Figure 2). Another more detailed circuit diagram can be found at: https://github.com/EwellNeuroLab/Discrimination-CA1/blob/main/8port%20Maze/Maze%20CircuitDesign.pdf):
      **CRITICAL:** Ensure the correct orientation of the rectifier diode to avoid damaging the circuit and the Arduino.
  b. Wire the breadboard for the light strip:
    i. Connect a MOSFET transistor to the breadboard.
    ii. Connect the gate terminal (G) of the MOSFET to the negative rail of the breadboard via a 1 kOhm resistor.
    iii. Connect the source terminal (S) of the MOSFET to the negative rail of the breadboard.
    iv. Repeat steps i-iii. for each light strip (x8).
  c. Wire the reward port breadboard for the emitter, sensor and LED:
    i. Connect a digital output pin to the breadboard for each reward port LED (x8) (Figure 1, olive green).
    ii. Connect an analog input pin to the breadboard for each reward port sensor (x8) (Figure 1, orange)
    iii. Connect a ground pin (GND) to the negative rail of the breadboard (blue).
    iv. Connect the 5V pin to the positive rail of the breadboard (Figure 1, red).
      ***Note:*** Both the solenoid valves and light strips require the 12 V voltage source.
3. Prepare cables for reward ports (Figure 3):
  a. Cut an ethernet cable with the desired length.
  b. Strip 7–10 cm of the outer jacket from the ethernet cable to expose the insulated wires.
  c. Untwist the wires and strip 2–3 cm isolation of each wire.
  d. Plug the ethernet cable in the RJ45 connector of the reward port.
  e. Map the connection between individual wires and the reward port.
    i. Turn on a multimeter and set it to continuity mode:
    ii. Touch the positive probe of the multimeter to the screw terminal labeled as *Emitter (−)* on the bottom of the *Bpod port interface* (Figure 3A, back of the reward port).
    iii. Touch the negative probe to the exposed wire of the ethernet cable one by one until a connection with *Emitter (−)* is found and label with an ‘E’ (Figure 3C).
    iv. Repeat steps ii-iii. For *LED (+)*, *S* and the two screw terminals of *VALVE* (Labels L and S and V, Figure 3C).
    v. As for *Emitter (+)*, take the positive probe and create a connection with the metal surface in the front of the *Bpod port interface* (Figure 3B, top left terminal).
    vi. Label each connected wire corresponding the part they connect to (e.g., IR emitter or sensor) and connect male header pins to them and cover with plastic housing (6 wires in total, Figure 3C).
    vii. Cut the rest of the two wires that did not make a connection.
  f. Repeat steps a-e. for each reward port (x8).

***Note:****Emitter (−), LED (−)* and *G* are connected to one common ground.

4. Connect the reward ports to the reward port breadboard (see Figures 1 and 3D):
  a. Connect the *Emitter (+)* wire to the positive rail of the breadboard (Figure 1, red).
  b. Connect the *Emitter (−)* wire to the negative rail of the breadboard (Figure 1, blue).
  c. Connect the *S* wire to an analog input pin on the breadboard (Figure 1, orange).
  d. Connect the *LED (+)* wire to a digital output pin on the breadboard (Figure 1, olive green).
  e. Connect the two VALVE cables to the two ends of the diode on the breadboard (Figure 1, magenta).
  f. Repeat steps a-e. for each reward port (x8).
5. Connect the light strips to the circuit (see Figures 1 and 2):
  a. Connect the drain terminal (D) of the MOSFET to the negative terminal of the light strip (Figure 1, cyan).
  b. Connect the positive terminal of the light strip to the positive rail of the breadboard (Figure 1, red).
  c. Repeat steps a-b. for each light strip (x8).

***Note:*** It is recommended to connect an additional light strip directly to the 12-volt battery pack as a visual help to the experimenter to determine whether the batteries are on or off.

### Setting up the Arduino code

**Timing: 30 min**

Custom-written Arduino codes should be downloaded from https://doi.org/10.5281/zenodo.17488388. Four codes are provided: two for habituation, one for the discrimination paradigm, and one for the generalization paradigm.

6. Connect the Arduino Mega 2560 to the computer.
7. Download the Arduino IDE from the Arduino website (https://www.arduino.cc/en/software/) and follow the instructions for installation.

***Note:*** Codes were tested on the 1.8.19 and 2.3.4 versions of the Arduino IDE.

8. Setting up the Arduino codes:
  a. Open the *ArduinoCode_Habituation_Day1.ino* code.
  b. Go to *Tools – Board* and select *Arduino Mega or Mega 2560.*
  c. Go to *Tools – Processor* and select *ATmega2560 (Mega 2560).*
  d. Go to Tools – Port and select the communications port for the Arduino (e.g., *COM3*).
  e. Take note of the digital output pin numbers of the light strip, reward port LED, valve and analog input for the IR sensor.
  f. If needed, change the pin list for the light strip (variable *LEDstrip*), reward port LED (variable *PortLED*), valve (variable *Valve*) and/or IR sensor (variable *IRSensor*).
  g. If needed, set the *RandomIN* variable to an analog input pin that is not connected to the maze (e.g., if A9 pin is not connected, set *RandomIN* to 9).
    ***Optional:*** Change how long the valves should stay open at each reward delivery (variable *ValveOpenTime*). The current 60–70 ms setting corresponds to approx. 10 μL.
  h. Repeat steps a-h. for the other three codes related to habituation day two and the discrimination/generalization task.
  i. In the discrimination/generalization codes, use the *PortIdx* variable to define which ports are rewarded (i.e., first element is the reward port in context A, second element is the reward port in context B).
    **CRITICAL:** It is important that the *RandomIN* variable is not set to an analog pin that is connected to the maze, otherwise trials will not be pseudo-randomized.

### Setting up the Bonsai workflow

**Timing: 30 min**

Custom-written Bonsai workflows should be downloaded from https://doi.org/10.5281/zenodo.17488388. Two workflows are provided: one for the habituation and for the discrimination/generalization task. Each workflow outputs three distinct files: the video (avi), X,Y position of the mouse body center (.csv) and timestamped events (e.g., nose pokes or trial start).

9. Download Bonsai-RX and follow the instructions for installation.
10. The following libraries need to be installed (*Tools – Manage Packages*):
  a. Bonsai.Audio.
  b. Bonsai.Vision.
  c. Bonsai.Vision.Design.
  d. Bonsai.Scripting.IronPython.
  e. Bonsai.Expressions.
  f. Bonsai.Reactive.
  g. Bonsai.Resources.
  h. Bonsai.IO.
  i. Bonsai.IO.Ports.
  j. Bonsai.Video.
  k. Bonasi.Dsp.
  l. Bonsai.FFmpeg.

***Optional:*** Bonsai.Miniscope

**Figure 4:**
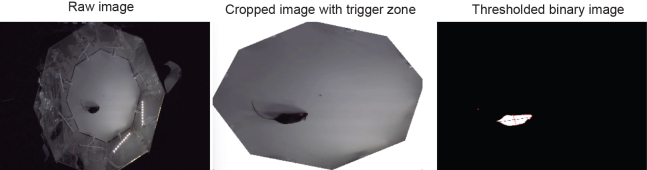
Schematics of real-time tracking of the mouse in Bonsai The raw video image (left) is cropped at the corners of the arena to capture the region of interest only (middle). Cropped image is then thresholded to detect the largest dark object in the field-of-view (rigth). Final coordinates correspond to the center of mass of the detected object.

11. Open the *Workflow Habituation.bonsai* file.
12. Set up the communication between the Arduino and Bonsai. If your communication port is COM3, skip this step.
  a. Open the *Init* node and set the *COM3* node *Name* and *PortName* to the communication port to which your Arduino is connected (e.g., COM4).
  b. Repeat step a. for the *SerialStringReadLine* and the *SerialWriteLine* nodes (located in *Trial – OpenPort* and *Trial – VisualCueOn*).
13. Define the square shaped random hidden trigger zone:
  a. Measure the pixel-to-cm ratio of your camera.
  b. Navigate to the *Trial – GetCoordinates - Int* node. Adjust the size of trigger zone in the *Value* field (measured in pixels).
  c. Double-click on the *Xmid* node and set the lower and upper boundaries to restrict where the trigger zone may appear (horizontal direction, measured in pixels).
  d. Repeat step c. for node to set the boundaries in the vertical direction.
14. Set up the real-time position tracking (Figure 2):
  a. Turn on the light source that will be used during experiments (e.g., light source of the camera).
  b. Place a mouse or dark object in the arena.
  c. Start the Bonsai workflow right-click on the *VideoCaptureDevice* node and select *Show Visualizer – Bonsai.Vision.Design.IplImageVisualizer* to visualize the view of the camera (Figure 4, left).
  d. Right-click on the window to display the pixel coordinates of the computer mouse.
  e. Type the coordinates of each corner of the arena in the *Regions* field of the *CropPolygon* node.
  f. Restart the Bonsai workflow and double-click on the *CropPolygon* node to visualize the cropped field of view (Figure 4, middle).
  g. Click on the *RangeThreshold* node and set the upper limits of the RGB colors in the *Upper* field (first three elements) such that only the mouse/object is detected (Figure 4, right).
  h. Readjust the cropping coordinates if dark objects near walls are detected.

***Note:*** Recommended light source is the built-in LED of a commercially available web camera (use the brightest mode). Camera should be centered above the arena (approx. 70 cm above).

***Note:*** When dark objects near the wall are detected, consider readjusting the cropping coordinates to ensure that the mouse will be tracked reliably during the experiments.

15. Set location for output files:
  a. Click on the VideoWriter node and set the path and file name in the FileName field.
  b. Repeat for the two *CsvWriter* nodes in the *SavePosition* and *Trial – ExperimentObserver* nodes respectively.
16. Repeat steps 12–15. for the *8port Workflow Training* file.
17. Set up the sound tone (*8port Workflow Training* only):
  a. Double-click on the *Audio Resources* node located in *Trial.*
  b. Add the path for the sound file in the pop-up window in the *FileName* field.
  c. Repeat steps a-b. within *Trial – ExperimentObserver* node.

### Setting up and cleaning the maze

**Timing: 20 min per day**

The following steps described must be completed every day before/after the experiment.

18. Mix 5 g of sucrose in 100 mL water until it is fully dissolved.
19. Fill up each 10 mL liquid container (syringe affixed to reward port) with the 5% sucrose.
20. Run the *OpenValves.ino* code until the liquid flows out on each reward port.

***Note:*** Residual liquid can trigger the IR sensor, therefore it is important to dry the reward ports before the next step.

21. Run the *TestMaze.ino* code to test the reward ports and lights:
  a. Verify that the reward port LEDs are switched on.
  b. Verify that the light strips on the walls are switched on.
  c. Trigger the IR sensor on each port using an object (e.g., a pen).
  d. When the IR sensor is triggered, the reward port LED turns off and a drop of sucrose is delivered.

***Note:*** It is recommended to test the reward ports and lights between each mouse.

22. At the end of the experiments, reward ports must be cleaned by flushing with alcohol and then flushing with distilled water:
  a. Run the *OpenValves.ino code* to release the remaining sucrose solution from the syringes.
  b. Fill the syringes up with 70% ethanol and flush it through the ports again running the *OpenValves.ino code.*
  c. Repeat step b using distilled water.
  d. Connect plastic tubing to a house vacuum to remove any residual liquid by placing the tube inside the syringe and then placing the tube over the metal cannula of the port (where the animal licks). ∗Note it is important to select a tubing with a gauge large enough to engulf the port cannula.

Run the *ShutOffMaze.ino* code to shut down the maze.

**CRITICAL:** Cleaning the ports with vacuum through the cannula is essential to maintain the reward ports. Otherwise, residual sugar can easily clog the solenoid valve.

## Key resources table

**Table:** 

REAGENT or RESOURCE	SOURCE	IDENTIFIER
**Chemicals, peptides, and recombinant proteins**		

Citric-acid	Sigma-Aldrich	C0759-1KG

**Experimental models: organisms/strains**		

C57BL/6 (male and female 3–5 month)	Charles River	027

**Software and algorithms**		

Custom code	this paper	https://doi.org/10.5281/zenodo.17488388
Arduino	Arduino	https://store-usa.arduino.cc/
Bonsai RX	Lopes et al^7^	https://bonsai-rx.org/

**Other**		

Laser-cut plexi-glass	Ponoko	https://github.com/EwellNeuroLab/Discrimination-CA1/blob/main/8port%20Maze/wall_design.eps
Arduino mega 2560 REV3	Arduino	A000067
Reward port assembly	Sanworks	1009
LED strip	Amazon	B0D9K4LM5T
1 kOhm resistor	E-Projects	10EP5121K00
MOSFET transistor	onsemi	RFP30N06LE-ND
Rectifier diode	BOJACK	1N4001

## Step-by-step method details

### Handling and habituation of animals

**Timing: 1 week (45–60 min per mouse per day)**

We used C57BL/6 mice (Charles River) at the age of 3–4 months at the beginning of the experiments. Habituation lasts for two days, in which mice are habituated to the maze and to the 5% sucrose reward. On day 1, sucrose is released from all ports when the mouse runs through the hidden trigger zone. On day 2, sucrose is released when mouse runs through the hidden trigger zone and pokes into a given port.

1. Handle mice for 3 to 4 days prior to habituation. If a major surgery precedes the experiment, allow one week of recovery.
2. Replace regular water with 2% citric-acid (CA) water^8^ two days prior to habituation:
  a. Mix 2 g of citric-acid in 100 mL water for each mouse.
  b. Weigh mouse before water is replaced to establish the baseline weight.

***Note:*** Monitor the weight of the mouse daily and maintain it over 85% of the baseline. If weight drops below the threshold, supplement additional regular water until the weight stabilizes over 85%.

3. Start habituation on the maze:
  a. Transfer the mouse to the behavioral room.
  b. Place the mouse in the center of the arena and close the door of the sound-proofing box.
  c. Run the Arduino code corresponding to the first/second day of habituation (see Table 1).
  d. Start the Bonsai workflow for habituation (see Table 1).
  e. After 45–60 min, stop the Bonsai workflow and transfer the mouse back to the cage.
  f. Transfer the output data into a dedicated folder for data storing.
  g. Wipe the floor, walls and ports with 50% alcohol and subsequently with water between each mouse. Alternatively the maze could be cleaned with fragrance free soap and water if plexiglass walls start to show deterioration.

**CRITICAL:** Some files can be overwritten if they are not transferred to a separate folder.

***Note:*** As the experiments are long, it is recommended to give some supplemental regular water (1–2 mL) to the mice before weekends, especially if experiments are not planned on the following day(s). Prepare fresh 5% sucrose daily.

**Table 1:** Codes and parameters for each phase of the experiment

Session	Arduino code	*TrainingBlock*	*MaxStrike*	Bonsai workflow
Filling up/empty mouse ports	OpenValves	N/A	N/A	N/A
Testing the mouse ports/light	TestMaze			
Shutting down the maze	ShutOffMaze			
Habituation day 1	ArduinoCode_Habituation Day1	N/A	N/A	Workflow Habituation
Habituation day 2	ArduinoCode_Habituation Day1	N/A	N/A	
Discrimination pretraining phase 1	ArduinoCode Discrimination_Training	800	10	Workflow Training
Discrimination pretraining phase 2		800	1	
Discrimination training/testing		8	1	
Generalization pretraining phase 1	ArduinoCode Generalization_Training	800	10	
Generalization pretraining phase 2		800	1	
Generalization training/testing		8	1	

Set the *TrainingBlock* and *MaxStrike* variables in the Arduino code according to the training phase.

### Pretraining on the discrimination paradigm

**Timing: 3–5 days (1 h per mouse per day)**

**Figure 5:**
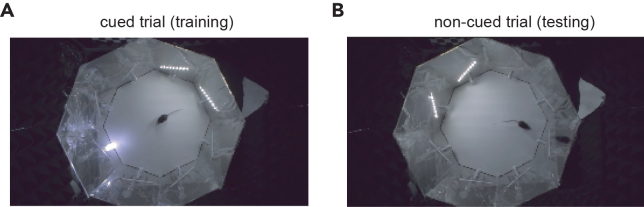
Difference between training and testing trials (A) An example of cued trial in which the LED serves as a visual guide to the correct port during reward retrieval. Pretraining and training trials employ cued trials. (B) An example of non-cued trial in which the mouse must find the reward location without the help of the LED. Performance during training/testing phase is calculated on these trials exclusively.

In the first phase of pretraining, mice are exposed to the two contexts for the first time. Mice are allowed to make mistakes during the reward retrieval phase. The trial ends when the mouse pokes to the correct port or after 1 min without poking. In the second phase, the trial is terminated if the mouse pokes in an incorrect port during the reward retrieval phase and the trial is considered as incorrect. In both phases, a lit LED on the rewarded port serves as a visual guide during reward retrieval (Figure 5A). To toggle between phases, modify the *TrainingBlock* and *MaxStrike* variables according to Table 1. Note that trials are pseudo-randomized (see setting up the Arduino code section).

4. Run the first and second phases of pretraining:
  a. Place the mouse in center of the arena and run the Arduino code with the proper settings and then the corresponding Bonsai workflow (see Table 1).
  b. From day 3, modify *TrainingBlock* and *MaxStrike* variables corresponding to the second phase (Table 1).
  c. The second phase ends when mouse performs at 70% or higher, or after 3 days.

### Training and testing on the discrimination paradigm

**Timing: 3–15 days (1 h per mouse per day)**

Both males and females were trained on the paradigm, and we did not find a statistical difference between the two sexes in terms of learning pace and performance. Each 24 non-cued testing trial block (Figure 5B) is interleaved with 8 cued training trials (Figure 5A). Performance is calculated on the testing trials only. Training ends when mouse reaches the 70% performance criteria. Modify the *TrainingBlock* and *MaxStrike* variables according to Table 1.

Note that each session starts with cued training trials and trials are pseudo-randomized in each block.

5. Conduct the discrimination training/testing experiments:
  a. Initialize the maze for the experiment (see step 3).
  b. After placing the mouse in the center of the arena, run the proper Arduino code and the corresponding Bonsai workflow (Table 1).
  c. Calculate the performance after each session.

***Optional:*** After mouse reaches the performance criteria, set the TrainingBlock variable in the Arduino code from 8 to 0 to assess how mouse performs without any training trials.

**CRITICAL:** Performance must be calculated on the testing trials only.

***Note:*** Some mice never learn the task, therefore it is recommended to terminate the experiments if a mouse does not reach criteria in 15 days.

### Training and testing on the generalization paradigm

**Timing: 2–6 days (1 h per mouse per day)**

We found no performance difference between males and females in the generalization paradigm. Mice do not need to be pretrained in the generalization task after they learned discrimination task first. Experiments end when mouse reaches the same 70% performance criteria.

6. Conduct the generalization training/testing experiments:
  a. Initialize the maze for the experiment (see step 3).
  b. After placing the mouse in the center of the arena, run the proper Arduino code and the corresponding Bonsai workflow (Table 1).
  c. The experiments are finished once mouse reaches the performance criteria. Replace the CA water with regular water.

***Note:*** If mouse is trained in the reversed order (generalization first, then discrimination) pretraining in the generalization should be included with the same timeline as described for discrimination (see step 4). When shifting from generalization to discrimination, pretraining is still necessary.

## Expected outcomes

The protocol provides instruction to build the open-source Discrimin8 maze and to implement the discrimination/generalization task. Mice learn the task within two to three weeks. Please see Tarcsay et al^1^ for visualization of learning curves. Bonsai RX is compatible with several open-source recording systems,^9^^,^^10^^,^^11^^,^^12^ allowing the experimenter to investigate physiological mechanisms during the task. Example workflows to implement recordings with Open Ephys or with the UCLA miniscope is provided on our GitHub page (https://github.com/EwellNeuroLab). The design of the maze (8 reward port and 8 light strip) provide flexibility for the experimenter to repurpose the maze and implement custom-designed tasks.

## Limitations

Several limitations should be acknowledged. We have only tested this paradigm on healthy mice. We believe with slight modification (i.e., positioning of reward ports etc.), the task would translate to rats. In the case of disease models, it is important that animals are able to forage and be maintained on water restriction. Another limitation involves over training; mice are trained on the discrimination paradigm typically for two weeks, that potentially results in the overtraining of the mice. Thus, the task may not be suitable to investigate physiological mechanisms with respect to behavioral performance. Second, some mice may not be able to learn the task, increasing the number of animals used in the study. Finally, reward ports and cables may wear out or fail over time, therefore periodic maintenance of the maze may be required (see troubleshooting section).

## Troubleshooting

### Problem 1

IR sensor of the reward port is triggered in the absence of nose poke or does not detect nose pokes. Related to step 21.

### Potential solution

Such issue implies a failure in the connection between the Arduino and the IR sensor and therefore the potential solutions are the following:

• Verify that other reward ports work. If not, verify that the emitter and ground cable on the reward port breadboard (Figure 1) is plugged properly.
• If only one IR sensor is faulty, verify that the ethernet cable is plugged in the reward port.
• Plug another ethernet cable (e.g., from the neighboring reward port) and test the IR sensor again. If the port works, the potential issue is the ethernet cable and must be replaced. If it still does not work, the IR sensor in the reward port may be faulty and it must be replaced.

### Problem 2

Solenoid valve does not release liquid. Related to step 21 (Setting up and cleaning the maze).

### Potential solution

We found that the solenoid valves may be clogged by the sucrose reward when the maze was not used for a longer period. Therefore, it is recommended to run the cleaning protocol weekly (see the setting up and cleaning the maze section), regardless of the experiments.

If the issue still occurs, download the *CleanPorts* code from our GitHub page. Running the script will try to open and close the valves periodically. Fill the liquid container syringes with water and let them run until the water is released by each port.

### Problem 3

Bonsai real-time tracking fails. Related to step 14 (Setting up the Bonsai workflow).

### Potential solution

In some cases, the camera – maze relative position may change. Therefore, first verify that the cropped image captures the floor of the arena and does not contain any blind spot. Moreover, inspect the view of the *RangeThreshold* node (see setting up the Bonsai workflow section and Figure 4). If the mouse cannot be seen, increase the threshold values. If ghost objects are detected, decrease the threshold values.

### Problem 4

The experiment requires mice with white fur. Related to step 14 (Setting up the Bonsai workflow).

### Potential solution

Bonsai tracking relies on the contrast between the mouse and the floor. In case of experiments with white mice, it is recommended to apply dark color Animal Marker (Braintree Scientific) on the back of the animal.

### Problem 5

The Bonsai workflow crashes during the recordings. Related to step-by-step method details.

### Potential solution

Ensure that the camera and other recording devices used during the experiment are connected. Furthermore, ensure that the local disk/external drive where the data is saved has sufficient free space. Lack of free space will result in a crash.

## Resource availability

### Lead contact

Further information and request for resources should be directed to and will be fulfilled by the lead contact, Laura A. Ewell (lewell@uci.edu).

### Technical contact

Technical questions on executing this protocol should be directed to and will be answered by the technical contact, Gergely Tarcsay (tarcsay.gergely@gmail.com).

### Materials availability

This study did not generate new unique reagents.

### Data and code availability

The code generated during this study is available on GitHub (https://doi.org/10.5281/zenodo.17488387).

## Acknowledgments

This work was funded by 10.13039/100000052NIH R01 1R01NS128222 (to L.A.E.). The authors thank the Bonsai RX community for sharing their knowledge on online forums that significantly contributed to the successful implementation of the workflows. Furthermore, the authors thank Dr. Brittney Boublil for naming the maze.

## Author contributions

Conceptualization, L.A.E. and G.T.; methodology, G.T.; software, G.T.; validation and investigation, G.T.; resources, L.A.E.; writing – original draft, G.T. and L.A.E.; writing – review and editing, G.T. and L.A.E.; supervision and funding acquisition, L.A.E.

## Declaration of interests

The authors declare no competing interests.
